# The immunoregulatory role of alpha enolase in dendritic cell function during Chlamydia infection

**DOI:** 10.1186/s12865-017-0212-1

**Published:** 2017-05-19

**Authors:** Khamia Ryans, Yusuf Omosun, Danielle N. McKeithen, Tankya Simoneaux, Camilla C. Mills, Nathan Bowen, Francis O. Eko, Carolyn M. Black, Joseph U. Igietseme, Qing He

**Affiliations:** 1 Department of Microbiology, Biochemistry, and Immunology, Morehouse School of Medicine, 720 Westview Drive S.W., Atlanta, GA 30310 USA; 2 Department of Biology, Clark Atlanta University, Atlanta, GA 30314 USA; 3 Centers for Disease Control & Prevention (CDC), Atlanta, GA 30333 USA

**Keywords:** Alpha enolase, ENO1, Chlamydia, Dendritic cells, Metabolism

## Abstract

**Background:**

We have previously reported that interleukin-10 (IL-10) deficient dendritic cells (DCs) are potent antigen presenting cells that induced elevated protective immunity against *Chlamydia.* To further investigate the molecular and biochemical mechanism underlying the superior immunostimulatory property of IL-10 deficient DCs we performed proteomic analysis on protein profiles from *Chlamydia*-pulsed wild-type (WT) and IL-10^−/−^ DCs to identify differentially expressed proteins with immunomodulatory properties.

**Results:**

The results showed that alpha enolase (ENO1), a metabolic enzyme involved in the last step of glycolysis was significantly upregulated in *Chlamydia*-pulsed IL-10^−/−^ DCs compared to WT DCs. We further studied the immunoregulatory role of ENO1 in DC function by generating ENO1 knockdown DCs, using lentiviral siRNA technology. We analyzed the effect of the ENO1 knockdown on DC functions after pulsing with *Chlamydia*. Pyruvate assay, transmission electron microscopy, flow cytometry, confocal microscopy, cytokine, T-cell activation and adoptive transfer assays were also used to study DC function. The results showed that ENO1 knockdown DCs had impaired maturation and activation, with significant decrease in intracellular pyruvate concentration as compared with the *Chlamydia*-pulsed WT DCs. Adoptive transfer of *Chlamydia*-pulsed ENO1 knockdown DCs were poorly immunogenic in vitro and in vivo, especially the ability to induce protective immunity against genital chlamydia infection. The marked remodeling of the mitochondrial morphology of *Chlamydia-*pulsed ENO1 knockdown DCs compared to the *Chlamydia*-pulsed WT DCs was associated with the dysregulation of translocase of the outer membrane (TOM) 20 and adenine nucleotide translocator (ANT) 1/2/3/4 that regulate mitochondrial permeability. The results suggest that an enhanced glycolysis is required for efficient antigen processing and presentation by DCs to induce a robust immune response.

**Conclusions:**

The upregulation of ENO1 contributes to the superior immunostimulatory function of IL-10 deficient DCs. Our studies indicated that ENO1 deficiency causes the reduced production of pyruvate, which then contributes to a dysfunction in mitochondrial homeostasis that may affect DC survival, maturation and antigen presenting properties. Modulation of ENO1 thus provides a potentially effective strategy to boost DC function and promote immunity against infectious and non-infectious diseases.

## Background

*Chlamydia trachomatis* genital infection is the most frequently reported bacterial sexually transmitted infection in the United States [1]. *C. trachomatis* is an obligate intracellular pathogen that causes a broad range of female reproductive diseases including pelvic inflammatory disease (PID), ectopic pregnancy and tubal factor infertility (TFI) [2]. In a recent study about 20% of PID and 29% of TFI in women aged 16–44 years was attributed to *C. trachomatis* infections [3, 4]. Preventive strategies proposed against *C. trachomatis* include increased screening with mass treatment [5, 6]. However, recent clinical and epidemiological data suggests that the vaccine strategy would be the most reliable and cost effective preventive method with the greatest potential impact in the control of *C. trachomatis* infections and its associated complications in the human population [7, 8]. However, there is no human vaccine available for *C. trachomatis* infections; the development of an effective vaccine against *C. trachomatis* has been a challenging task due to the incomplete understanding of the complex immunological processes associated with chlamydial infection.

Studies using mouse models of genital chlamydial infection have shown that infection causes dendritic cells (DCs) to undergo maturation and strongly up-regulate major histocompatibility complex (MHC) antigens, and costimulatory molecules such as cluster of differentiation (CD) 40, CD80 and CD86, which are crucial for effective activation of the CD4^+^ T helper cell type 1 (Th1) cell response, especially the CD4 T cells and interferon (IFN) -γ production required for host defense [9–11]. Current vaccine efforts are focusing on antigen delivery and immunomodulatory strategies to induce protective immunity and at the same time prevent detrimental immunopathologies.

DCs are potent, professional antigen-presenting cells that play very import role in primary humoral and T-cell-mediated immune responses. It is gradually becoming apparent that different stages of immune cell activation coincide with, and are underpinned by, different types of cellular metabolism that are tailored towards the bioenergetic and biosynthetic needs of these cells. Emerging data have recently demonstrated the contribution of cellular metabolic pathways to the ability of immune cells to sense their microenvironment and thereafter alter their functions [12–15]. Distinct changes in the microenvironment induce a spectrum of inducible and reversible metabolic programs that might be necessary in functional immune cell activation/polarization phenotypes. For example, after exposure to Toll-like receptor agonists, DCs undergo a metabolic transition from oxidative phosphorylation (OXPHOS) to aerobic glycolysis, which is required to meet the increased biosynthetic and bioenergetic demands of activated DCs specifically by funneling metabolites into pathways for lipid and protein synthesis [14, 16]. Cytokines such as IL-10 and IFN-γ are key regulators of immune responses whose actions are mediated via cellular process that include active metabolism. As a negative immune-modulator, IL-10 suppresses inflammatory immunostimulation by acting on both regulatory and effector immune cells but the molecular mechanisms are not completely understood. Interestingly, IL-10 deficient DCs exhibit a high efficiency in chlamydial antigen presentation and the induction of a high frequency of specific immune effectors that protect against chlamydial disease [17]. Proteomics and functional immunologic analyses revealed that the efficient APC function of IL-10 deficient DCs coincided with rapid maturation, and expression of high levels of co-stimulatory and metabolic molecules, including Leucine/glutamic acid/lysine protein 1 (LEK1), Vimentin, Arginase-1, Fatty acid binding protein and ENO1. This suggests that IL-10 deficient DCs are highly effective cellular vaccines that possess certain immunomodulatory features that provide us a suitable immunotherapeutic platform to better define the immunological and biochemical determinants and conditions for inducing adequate and long-term immunity against *Chlamydia*-induced tubal pathologies [18, 19]. Such immunostimulatory features can also be applied to improve vaccine delivery and elicitation of protective immunity against other infectious and non-infectious diseases in general [17, 20].

Proteomics data showing that ENO1 was significantly increased in IL-10^−/−^ DCs suggested that it may play a role in the immunostimulatory properties of the DCs. ENO1 is a glycolytic enzyme which is expressed in cytoplasm, nucleus, and on the surface of many eukaryotic cells, such as stimulated hematopoietic (neutrophils, B & T lymphocytes and monocytes), epithelial, neuronal and endothelial cells. Its functions are related to its subcellular location, and ENO1 catalyzes the dehydration of 2-phosphoglycerate to phosphoenolpyruvate [21, 22], which is an important step in ATP generation through substrate-level phosphorylation. ENO1 has been found to play other roles in inflammation, tumor suppression, monocyte and mast cell differentiation [21, 22]. Disruption of glycolysis through the reduced expression of ENO1 has recently been linked to NLR family pyrin domain containing 3 (NLRP3) activation [23], and we have previously shown that NLRP3 inflammasome assembly was suppressed in IL-10^−/−^ DCs [18]. In this study, we further analyze the molecular basis of the potent immunostimulatory action of IL-10 DC focusing on the contribution of ENO1 to immunostimulation. We used siRNA technology to knockdown ENO1 in DCs and evaluated the maturation, activation, and antigen presenting functions of DCs in vivo and in vitro. In addition, we also investigated the remodeling of DC mitochondria, which controls metabolic programs and therefore regulates DC function. We provide evidence for the role of ENO-1 in the potent immunostimulatory function of IL-10 deficient DCs, which involves the regulation of DC metabolism, DC mitochondrial modeling, DC survival and antigen presentation function.

## Methods

### Chlamydia stocks

Stocks of *C. muridarum*, provided by the Molecular Pathogenesis Laboratory of the Division of Scientific Resources, Centers for Disease Control and Prevention were used for stimulating DCs. They were prepared by propagating elementary bodies (EBs) in McCoy or HeLa cells. The cell lines were maintained in minimum essential medium (MEM) supplemented with 2 mM L-glutamine, 10% heat-inactivated FBS, 1 mM sodium pyruvate, 0.5% fungizone, and 1.0% penicillin/streptomycin (100U/mL; 100 μg/mL) in an incubator at 37 °C in 5% CO2. *Chlamydia* stock titers were expressed as inclusion forming units (IFU) per milliliter.

### Animals

Six-week-old female WT mice (C57BL/6 J background) were purchased from The Jackson Laboratory (Bar Harbor, ME). The mice were fed with food and water ad libitum and maintained in laminar flow racks under pathogen-free conditions of 12 h of light and 12 h of darkness. The animal use protocols described in this proposal have been approved by the Institutional Animal Care and Use Committee of Morehouse School of Medicine (MSM-IACUC) and follow approved federal guidelines.

### DC isolation and culture

Immature DCs were isolated from bone marrow collected from the femurs of WT mice and cultured by plating the cells in 100 cm^2^ dishes using DC culture media containing complete RPMI 1640 medium, FBS, HEPES, glutamine, nonessential amino acids, sodium pyruvate, gentamicin, mouse recombinant interleukin-4 (IL-4) and granulocyte-macrophage colony-stimulating factor (GM-CSF) (Gemini Bioproducts, Sacramento, CA). The cells were cultured in an incubator at 37°Celsius in 5% carbon dioxide air, replacing with fresh media on day 3 and transferring cells to new dishes on day 5. After 5 days in culture, the cells were characterized as loosely adherent mononuclear cells and further purified as CD11c expressing DCs by using the Pan Dendritic Cell Isolation Kit from Miltenyi Biotec (San Diego, CA) [24]. Cells were then ready to be used in the experiments as described.

### Lentiviral transfection of ENO1 small interfering RNA in dendritic cells

For ENO1 knockdown studies, a siRNA vector against mouse ENO1containing H1 promoter (piLenti-siRNA-GFP) was purchased from Applied Biological Materials (Richmond, BC, CA). The siRNA vector has an independent open reading frame of green fluorescent protein (GFP). The GFP-positive cells were monitored as a marker for transfection. The target sequences were;

- 5′-ACTGTTGAGGTCGATCTGTACACCGCAAA-3′,
- 5′-GCCCTAGAACTCCGAGACAATGATAAGAC-3′,
- 5′-GCGCCTGCTCTGGTTAGCAAGAAAGTGAA-3′, and
- 5′ -GCCACCAATGTGGGTGATGAGGGTGGATT-3′.

Controls used were the scramble siRNA vector target sequence was 5′-GGGTGAACTCACGTCAGAA-3′ and WT DCs without siRNA vector. DCs were incubated overnight in 6 well plates with complete media without IL-4 and GM-CSF. Cells were transfected with 1 μg of siRNA vector and seeded at a density of 0.3 × 10^6^ cells per well in complete media containing polybrene (8 μl/ml) and after 48 h, cell viability was checked with trypan blue. This experiment was repeated 3 times.

### Proteomics assay

In the proteomic analysis of WT and IL-10^−/−^ DCs, DCs were pulsed with Chlamydia for 0, 2, 4 and 8 h. Proteins were then extracted from the Chlamydia pulsed DCs using a Bio-Rad protein extraction kit (Bio-Rad, Hercules, CA) and then cleaned up with a 2-D Clean up Kit (Bio-Rad, Hercules, CA), following the manufacturer’s protocol. Protein concentration was determined using 2D Quant Kit (GE Healthcare, Piscataway, NJ). Samples were labeled with Cy5 (Red-IL-10^−/−^DCs) and Cy3 (Green-WT DCs) respectively, then mixed together in a rehydration buffer and subjected to two-dimensional fluorescence differential gel electrophoresis analysis (2D-DIGE). Yellow color indicated the expression of the same protein by both WT DCs and IL-10^−/−^ DCs, while, green color indicated proteins expressed at a higher level in WT DCs compared to IL-10^−/−^ DCs. Red color indicated proteins in IL-10^−/−^ DCs that were over-expressed compared to WT DCs. The spots corresponding to differentially expressed proteins were digested and analyzed by nanocapillary LC–MS/MS (Xevo G2 Tof, 210 Waters, Milford, MA). Protein candidates were identified using automated database search against the NCBI database using MASCOT Daemon software (Matrix Science Inc., Boston, MA). This experiment was repeated 3 times.

### Western blot analysis

Lysates from *Chlamydia* pulsed and nonpulsed WT, IL10^−/−^ and ENO1 knockdown DCs were prepared by homogenization in lysis buffer supplemented with 1 mmol/L phenylmethylsulfonyl fluoride and protease inhibitor cocktail. 20 μg protein of *Chlamydia* pulsed and nonpulsed WT, IL10^−/−^ and ENO1 knockdown DCs lysates were loaded onto 4–20% TGX gradient gel (Bio-Rad, Hercules, CA) and run for 1 h. Proteins were then transferred onto nitrocellulose paper (Bio-Rad, Hercules, CA). After 1 h, the blots were washed, blocked with 5% milk, and then incubated with desired primary monoclonal antibody against ENO1 raised in rabbit (Abcam, Cambridge, MA), overnight at 4 °C. Goat anti-rabbit Horseradish peroxidase (HRP)-conjugated secondary antibodies (Southern Biotech, Birmingham Al) were added for 1 h at room temperature, and then the blots were developed using Clarity Western enhanced chemiluminescence (ECL) substrate (Bio-Rad, Hercules, CA). Viewing and quantification was analyzed using ImageQuant LAS 4000 (GE Healthcare, Pittsburgh, PA). The experiment was repeated three times.

ENO1 has been shown to be associated with cytoskeletal proteins in cells [25], so in order to get a true and definite picture of protein expression in DCs we used the Cy5 Total Protein Normalization method [26]. In this method, chlamydia pulsed and nonpulsed WT and ENO1 knockdown DC cell lysates were stained for total protein with Amersham WB CY5 antibody (GE Healthcare, Pittsburgh, PA) for 30 min at room temperature and ran on TGX gels (Bio-Rad, Hercules, CA) for 1 h. Proteins were then transferred in seven minutes onto PVDF membrane (Bio-Rad, Hercules, CA) using the Trans-Blot Turbo Transfer System (Bio-Rad, Hercules, CA). The blots were washed, then blocked with 5% milk and incubated with primary antibody; PDH-Eα (mouse monoclonal), TOM20 (rabbit polyclonal), ANT1/2/3/4 (rabbit polyclonal) and GAPDH (rabbit polyclonal) (Santa Cruz Biotech., Dallas, TX.) overnight at 4 °C. Subsequently, blots were washed five times in TBS/Tween followed by incubation with Cy3 anti-mouse and anti-rabbit secondary antibody (GE Healthcare, Pittsburgh, PA). Blots were washed and developed using Western ECL substrate and the fluorescence was viewed using the LAS 4000 Gel Doc System (GE Healthcare, Pittsburgh, PA). Normalization was carried out with the ImageQuant TL Software 8.1 (GE Healthcare, Pittsburgh, PA). The images were saved as Tiff files. This experiment was repeated 3 times.

### Confocal microscopy

WT and IL-10^−/−^ DCs (1 × 10^6^ cells/ml) were pulsed with Chlamydia (MOI of 5) for 1 and 2 h, and then fixed for 5 min at room temperature in PBS solution containing 4% formaldehyde/ 0.01% glutaraldehyde. Samples were washed twice in cold 1X PBS and the Fc receptors were blocked with Fc blocker. DCs were incubated with Anti-ENO1 (Abcam, Cambridge, MA) for 1 h at 4 °C and washed twice with 1X PBS. DRAQ5 and Alexa Fluor 488 bound secondary antibodies (Jackson ImmunoResearch Laboratories, West Grove, PA) were then added for 1 h at 4 °C. DCs were washed three times and resuspended in wash buffer overnight at 4 °C. Confocal images were obtained on the Zeiss 510 VIS 234 Confocal Microscope (Carl Zeiss Microscopy, GmbH). Images were taken from different fields on each plate. Quantitative colocalization analysis was used in analyzing the data (ImageJ Software, NIH, USA). We repeated this experiment 3 times.

### Flow cytometry

WT and ENO1 knockdown DCs pulsed with *C. muriduram* at MOI of 5 were resuspended in PBS containing 2% FBS (wash buffer) and washed and incubated for 10 min at 4 °C with 5 μg/mL Fc blocker. The cells were incubated with fluorescein isothiocyanate (FITC)-, phycoerythrin (PE)-, or allophycocyanin (APC)-conjugated antibodies against surface markers CD40, CD80, CD86, MHC II, and toll like receptor 4 (TLR4) (BD Pharmingen, San Jose, CA) for 1 h at 4 °C. Thereafter, the cells were washed 3 times and resuspended in wash buffer, and then filtered into 5 ml FACS tubes, and the flow data was acquired on a BD Accuri C6 and analyzed using BD Accuri C6 software (BD, Franklin Lakes, NJ), and for each sample, at least 100,000 events were collected, and the experiment was replicated 3 times.

### Apoptosis assay

The level of apoptosis in chlamydia pulsed and nonpulsed WT and ENO1 knockdown DCs was determined using Annexin V and 7-AAD flow cytometry (Biolegend, San Diego, CA). The cells were then collected at time 0 and 2 h after infection and washed with cold BioLegend Cell Staining Buffer twice and then resuspended in Annexin V binding buffer. Five microliters of fluorescein isothiocyanate (FITC)-Annexin V and 5 μl of 7-amino-actinomycin D (7-AAD), viability staining solution were added to 100 μl cell suspensions and incubated in the dark at room temperature for 15 min. 400 μl of binding buffer was added to the sample and the mixture was vortexed and then analyzed by flow cytometry using a guava easyCyte 8HT (EMD Millipore, Billerica, MA). For each sample, at least 100,000 events were collected. Data was analyzed with guavaSoft 2.7 (EMD Millipore, Billerica, MA).

### Cytokine assay

WT and ENO1 knockdown DCs were plated at 1 × 10^6^ cells/ml in culture media and then DCs pulsed with *C. muriduram* at MOI of 5 for 1 to 2 h. Culture supernatants were collected and the amount of cytokines produced was determined using a Bio-Plex Pro Mouse Cytokine Assay kit (Bio-Rad, Hercules, CA) in accordance with the manufacturer’s protocol. The mean and SD of all replicate cultures were calculated. The experiment was repeated three times.

### T-cell proliferation assay

Lymphocytes were obtained from spleens of WT mice intravaginally infected *with Chlamydia* using a 40 μm filter and syringe plunger and suspended in PBS solution. The CD4^+^ T cells were then purified using the MACS mouse Pan T Cell Isolation Kit (mouse) (Miltenyi Biotec, Inc, San Diego, CA). To assess the antigen-presenting function of WT and ENO1 knockdown DCs, 1 × 10^5^ γ-irradiated DCs were co-cultured with 2 × 10^5^ purified T cells in the presence or absence of UV-inactivated *C. muriduram* (MOI of 5) in 96-well tissue culture plates for 5 days. The amounts of IL-1β, IL-1α IL-5, IL-9, IL-10, IL-13, IL-17A, and RANTES in the culture supernatants were measured using the Bio-Plex Pro Mouse Cytokine 23-Plex multiplex array according to the manufacturer’s guidelines (Bio-Rad) using a Luminex machine. The T cell proliferation was detected using a spectrophotometer set at 450 nm following the XTT Cell Viability Kit protocol (Cell Signaling Technology, Danvers, MA). The concentration of cytokine in each sample was obtained by extrapolation from a standard calibration curve generated simultaneously. The mean and SD of all replicate cultures were calculated. The experiment was repeated two times.

### Pyruvate assay

Supernatant from DCs pulsed with *C. muriduram* at MOI of 5 (0, 0.5, 1, 2, and 4 h) were diluted with pyruvate assay buffer and the pyruvate assay was carried out following the manufacturers’ protocol (Eton Bioscience, San Diego, CA). Final measurements of pyruvate concentration were done at 570 nm on a microplate reader. The mean and SD of all replicate cultures were calculated. The experiments were repeated three times.

### Adoptive transfer of DCs and assessment of protective immunity in vivo

IL-10KO, WT and ENO1 knockdown DCs pulsed with *C. muriduram* at MOI of 5 were adoptively transferred through intravenous tail injection (2.5 × 10^7^ cells/mouse) into 6–8 weeks old WT mice (*n* = 4 per group) pre-treated with Depo Provera (Pfizer Inc., NY, NY), which is used to synchronize the estrous cycle. Mice were infected intravaginally 1 week later with 20 μl of 1 × 10^5^ IFUs of *C. muriduram,* which we have shown to cause disease pathology in mice [17]*.* The course of the infection was determined by periodic swabbing (every three days for two weeks and later once a week for two weeks). Chlamydia culture confirmation kit (Bio-Rad, Hercules, CA) was used to determine the bacteria IFUs. The experiment was repeated three times.

### Transmission electron microscopy (TEM)

WT and ENO1 knockdown DCs were pulsed with *C. muriduram* at MOI of 5 for 2 h in 24-well tissue culture plates. The cells were then washed and fixed glass slides using 2.5% (w/v) glutaraldehyde/0.1 M cacodylate buffer for 2–6 h at 25 °C. DCs were then mounted and cut into 200 mm slices with a Vibratome (EM Corp., Hatfield, PA), and post fixed in aqueous osmium tetroxide. Fixed DC slices were dehydrated using graded ethanol and propylene oxide, and embedded in Polybed 812 resin (Polysciences, Inc., Warrington, PA). Thin sections (80 nm) were then cut with a diamond knife and stained with 5% uranyl acetate and Reynold’s lead citrate. A JEOL 1200EX transmission electron microscope (JEOL USA, Inc., Peabody, MA) was used to examine mitochondrial changes. The experiment was repeated three times.

### Statistical analysis

The data derived from different experiments was analyzed and compared by performing a 1- or 2-tailed *t*-test. The relationship between the diverse experimental groups was evaluated by analysis of variance (ANOVA) (Microsoft Excel 2015, Redmond, WA; GraphPad Prism, La Jolla, CA). Statistical significance was determined at *P* < 0.05.

## Results

### Upregulation of ENO1 expression in DCs following Chlamydia pulse

**Fig. 1:**
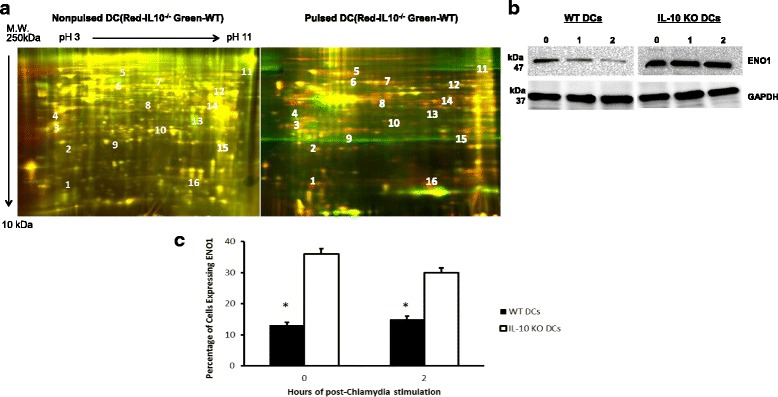
Dynamic Distribution of ENO1 in DCs. **a** Dendritic Cell lysates were extracted from *Chlamydia* pulsed or nonpulsed WT DCs and IL-10^−/−^ DCs. The Cy5 represents *red dye* and Cy3 represents *green dye*. The *red color* indicates high expression of protein in IL-10^−/−^ DCs while the *green color* indicates high expression of protein in WT DCs. The *yellow color* indicates that the protein expression is the same for WT and IL-10 ^−/−^ DCs. **b** Western blot analysis of WT DCs and IL-10^−/−^ DCs pulsed for 0 h, 1 h, and 2 h with *Chlamydia*. This result shows that ENO1 expression is decreased in WT DCs pulsed with *Chlamydia*. However, ENO1 expression is highly expressed in IL-10^−/−^ DCs pulsed with *Chlamydia*. **c** In addition, we compared the amount of Chlamydia pulsed and nonpulsed WT and IL-10^−/−^ DCs expressing ENO1 using confocal microscopic analysis. There was a significant difference in the amount of ENO1 expressing DCs in IL-10^−/−^ DCs and WT DCs (*p* < 0.05). * Denotes statistical significance. We repeated this experiment 3 times

Total protein extracted from *Chlamydia*-pulsed or nonpulsed WT and IL-10^−/−^ DCs were labeled with Cy5 (red, IL-10^−/−^ DCs) and Cy3 (green, WT DCs) and subjected to two-dimensional fluorescence differential gel electrophoresis (2D-DIGE) analysis and LC-MS/MS to identify differentially expressed molecules. Here we show a representative protein profile map revealing that some proteins were differentially expressed by IL-10^−/−^ DC compared with WT DC (Fig. 1a). At least three notable categories of differentially expressed proteins were identified: 1) proteins involve in cell metabolism (i.e., ENO1, Arginase-1, Isocitrate dehydrogenase [NADP], Triosephosphate isomerase, and Fatty acid-binding protein). 2) Proteins that are members of the cell cytoskeletal network (i.e., Coactosin-like protein, Macrophage capping protein, and Vimentin). 3) Proteins involved in the process of protein folding (i.e., Peptidyl-prolyl cis-trans isomerase A and Heat shock cognate 71 kDa protein). To rule out the possibility that we were working with normally overly expressed proteins, we evaluated the biological significance of the proteins, when we evaluated the metabolism related proteins by Western Blotting, ENO1 which is a key glycolytic enzyme was remarkably upregulated in IL-10^−/−^ DCs compared to WT DCs (Fig. 1b). Moreover, we analyzed the percentage of IL-10^−/−^ DCs expressing ENO1 using confocal microscopy (Fig. 1c). The results confirmed the upregulation of ENO1 as well as the dynamic distribution of ENO1 in IL-10^−/−^ DCs after pulse with *Chlamydia*.

### ENO1 Knockdown with ENO1-set siRNA Lentivector to study the role of ENO1 in DC function

**Fig. 2:**
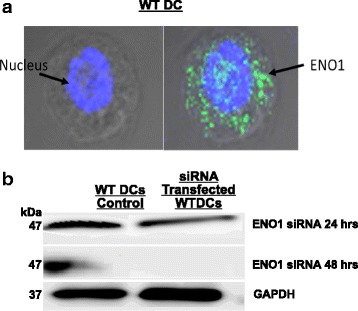
ENO1 Knockdown with ENO1-set siRNA Lentivector. **a** The confocal microscopy shows that that ENO1 is expressed most abundantly in the cytoplasm of the DCs. **b** ENO1 siRNA was used to knockdown ENO1 in WT DCs at 0 h, 24 h and 48 h. Scramble siRNA was used as a control for ENO1 knockdown confirmation. ENO1 is expressed in siRNA Scramble control and ENO1 siRNA at 0 h and 24 h respectively. ENO1 expression was completely knocked down after 48 h of transfection. Therefore, we used WT cells, which have been transfected with ENO1 siRNA for 48 h throughout our experiments to analyze the effects of ENO1 on DC function. We repeated this experiment 3 times

Rapid and elevated T cell response is required for the clearance and establishment of long-term immunity against certain infectious and non-infectious diseases. Therefore, a better understanding of the functional aspects of the differentially expressed proteins in chlamydia-pulsed IL-10^−/−^ DCs may furnish targets for modulating antigen presentation for enhanced T cell response against intracellular microbial pathogens and tumors. Using genetically engineered specific gene knockout systems and immunological and biochemical blockers, we analyzed the effect of siRNA-mediated knockdown of ENO1 on DC functional integrity. The distribution of ENO1 in WT DCs is localized in both the nucleus and the cytoplasm, although it is more prominent in the latter, since the location of ENO1 determines its function (Fig. 2a). ENO1 expression was completely knocked down after 48 h of lentivector transfection. The control oligomer with a scrambled sequence, as described in the Methods section, did not show any reduced ENO1 expression (Fig. 2b).

### ENO1 knockdown DCs have altered metabolism and mitochondrial morphology

**Fig. 3:**
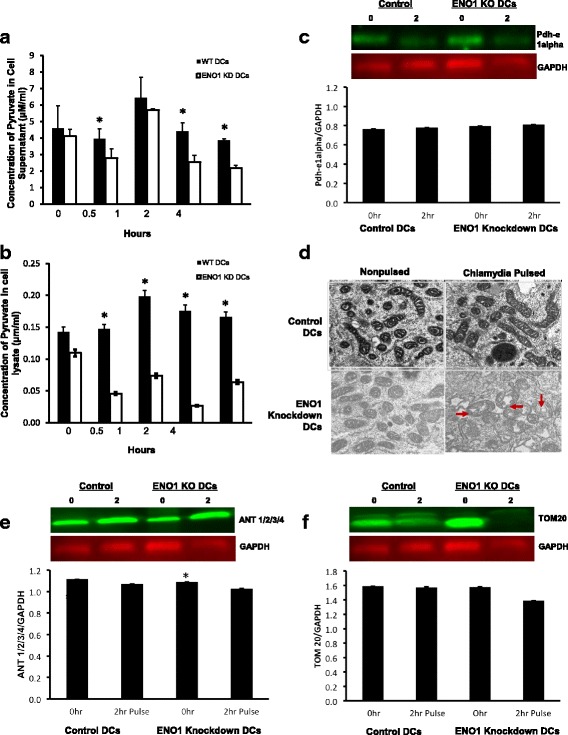
ENO1 knockdown DCs have altered metabolism and mitochondrial morphology. **a & b** Pyruvate Concentration in *Chlamydia*-pulsed Dendritic Cells. Pyruvate concentration was obtained in DC lysates and supernatant. Pyruvate concentration was measured using a spectrophotometer. Pyruvate concentration was determined after pulsing with *Chlamydia* at different time points for 0, 0.5, 1, 2 and 4 h. The concentration of pyruvate was significantly higher in WT cells compared to ENO1 knockdown DCs (*p* < 0.05). * Denotes statistical significance. This experiment was repeated 3 times. **c** Pyruvate dehydrogenase expression in Chlamydia-pulsed WT and ENO1 knockdown DCs. The total protein was detected using Cy5 dye and the protein of interest, in this case pyruvate dehydrogenase, was probed using Cy3 secondary antibody from the Amersham System (GE Healthcare, Pittsburgh, PA). Proteins were normalized using ImageQuant Reader (GE Healthcare​, Pittsburgh, PA). The ratio of Cy3/Cy5 indicates the differentially expressed proteins. This experiment was repeated 3 times. **d** TEM analysis of the WT and ENO1 knockdown DCs. The results indicate that there are morphological differences in the mitochondria of Chlamydia pulsed and nonpulsed WT and ENO1 knockdown DCs using TEM. This depicts the remodeling of the mitochondria to tubular and fused, with tight cristae in the WT DCs and to more circular, swollen and fissed with loose cristae in ENO1 knockdown DCs. **e** & **f** Expression of mitochondrial associated proteins. The blots shown are proteins associated with mitochondrial function. ANT 1/2/3/4 and TOM 20 were differentially expressed in Chlamydia pulsed ENO1 knockdown DCs. * denotes statistical significance. This experiment was repeated 3 times

The metabolic transition from oxidative phosphorylation (OXPHOS) to aerobic glycolysis upon exposure to microbial pathogens as the source of antigens is required for DC maturation and function. We therefore investigated the hypothesis that the suppression of ENO1 will interrupt this metabolic switch, suppress DC maturation and function upon exposure to *Chlamydia.* To confirm this hypothesis, we measured the production of pyruvate in DCs pulsed or nonpulsed with *C. muridarum*. DCs were pulsed at different time points and both the intracellular production of pyruvate and secretion of pyruvate were measured. The result showed that the secretion of pyruvate was significantly reduced in ENO1 knockdown DCs pulsed with *Chlamydia* at 0.5, 2, and 4 h (*P* = 0.024, 0.031, and 0.021, respectively) compared to *Chlamydia* pulsed WT DCs that had their ENO1 intact (Fig. 3a). The intracellular pyruvate production was also significantly decreased in ENO1 knockdown DCs at 0.5, 1, 2, and 4 h (*p* ≤ 0.05) (Fig. 3b). We then investigated if the knockdown of ENO1 affected other enzymes involved in pyruvate metabolism further downstream. The expression of pyruvate dehydrogenase, which is involved in the conversion of pyruvate to acetyl CoA initiating the TCA (citric acid) cycle, was evaluated. Our results showed that pyruvate dehydrogenase expression was essentially indistinguishable in *Chlamydia*-pulsed and non-pulsed ENO1 knockdown DCs and WT DCs (Fig. 3c). These results suggest that the absence of ENO1 slowed the rate of production of pyruvate after pulsing the DCs with *C. muridarum* and that pyruvate dehydrogenase is not involved in this process.

Mitochondria are central to cellular energetics, metabolism, and regulation. The shape of the mitochondria and remodeling of the mitochondrial cristae instructs cell metabolic adaptation. To determine if the knockdown of ENO1 also influenced the mitochondrial phenotype we proceeded to perform transmission electron microscopy (TEM). Alteration of mitochondrial shape as well as cristae remodeling were observed in *Chlamydia*-pulsed ENO1 knockdown DCs. There appeared to be elongated mitochondria with tubular network-like cristae in nonpulsed WT, ENO1 knockdown DCs and *Chlamydia-*pulsed WT DCs, whereas, we observed fragmented mitochondria morphology with widened intermembrane space along with short and stumpy cristae in *Chlamydia-pulsed* ENO1 knockdown DCs (Fig. 3d). This led us to investigate whether ENO1 knockdown influenced the expression of some mitochondrial-associated proteins (Tom20 and ANT1/2/3/4). Western blot analysis showed a differential expression of TOM20 and ANT 1/2/3/4 in *Chlamydia-*pulsed ENO1 knockdown DCs (Fig. 3e). These data suggest that in suppression of ENO1 expression leads to mitochondrial cristae remodeling which is coincident with metabolic reprogramming in *Chlamydia-*pulsed *D*Cs.

### ENO1 affects DC apoptosis

**Fig. 4:**
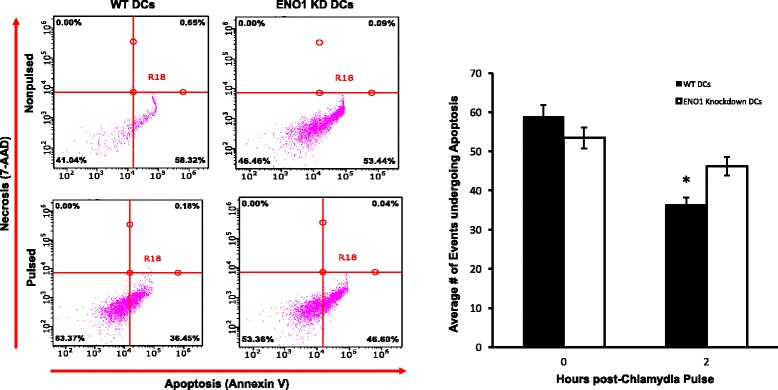
ENO1 affects DC apoptosis. Flow Cytometry analysis was used to determine the rate of apoptosis in Chlamydia pulsed and nonpulsed WT and ENO1 knockdown DCs. DCs were stained with CD11c PE-CY7 stain and gated for further analysis. The DCs then were stained with Annexin V and 7-AAD. The *Green fluorescence* is FITC while the *Red fluorescence* is 7-AAD. Quadrant 4 (*lower right*) indicates cells undergoing apoptosis. The *graph* shows the average number of cells undergoing apoptosis and this has been depicted in their percentages. * Denotes statistical significance. This experiment was repeated 3 times

DC survival is crucial for the APC function and we have previously reported that IL-10 deficient DCs resist apoptosis [18]. We therefore hypothesized that ENO1 upregulation may play a role in DC resistance to apoptosis and promotion of cell survival following exposure to *Chlamydia* antigen. Results from analysis of ENO1 knockdown DCs apoptosis using annexin V and 7-AAD showed that *Chlamydia*-pulsed ENO1 knockdown DCs were more apoptotic than the WT DCs (*p* < 0.05) (Fig. 4). This indicates that ENO1 is an essential anti-apoptotic molecule in DCs during chlamydia infection and that IL-10 deficiency that upregulates ENO1 in DCs decreases susceptibility to apoptosis.

### ENO1 regulates DC maturation and activation

**Fig. 5:**
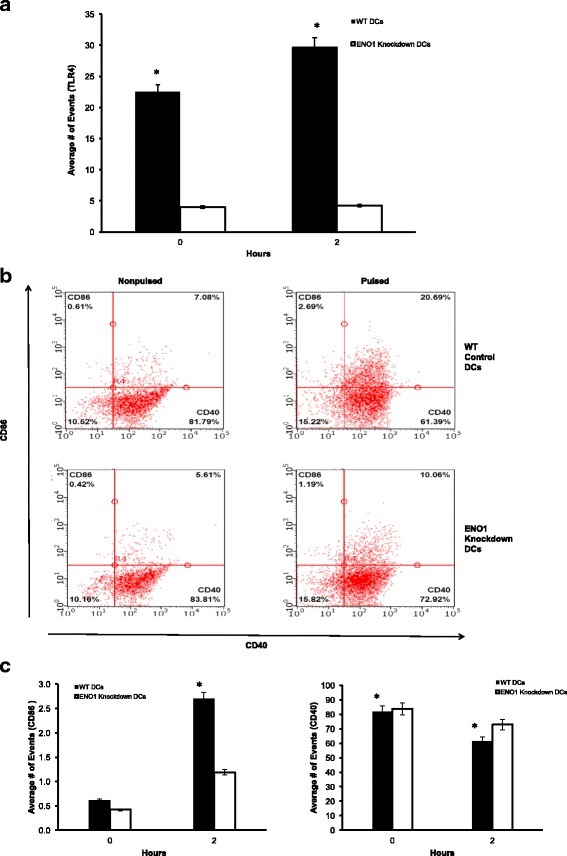
ENO1 regulates DC maturation and activation (1). **a** Flow cytometry analysis using BD Accuri C6 analyzer was used to determine TLR4 expression. DCs were stained with CD11c PE-CY7 stain and gated for further analysis. Cells were then stained with FITC conjugated with TLR4. TLR4 expression was significantly lower in Chlamydia pulsed ENO1 knockdown DCs in comparison to Chlamydia pulsed WT DCs. **b** Flow Cytometry analysis was also used to determine the population of cells with the maturation and activation markers CD40 and CD86 expression in DCs. Cells were stained with FITC conjugated CD40 and PE Conjugated CD86. Samples double stained with PE and FITC were compensated. Green-B-Fluorescence indicates FITC staining and Yellow-B Fluorescence indicates PE staining. **c** The graph shows that there were significant differences between WT and ENO1 knockdown DCs (*p* < 0.05). The expression of CD86 was lower in Chlamydia pulsed ENO1 knockdown DCs compared to WT DCs. While, CD40 expression increased in Chlamydia pulsed ENO1 knockdown DCs. Experiment was repeated 3 times for each sample. * Denotes statistical significance

**Fig. 6:**
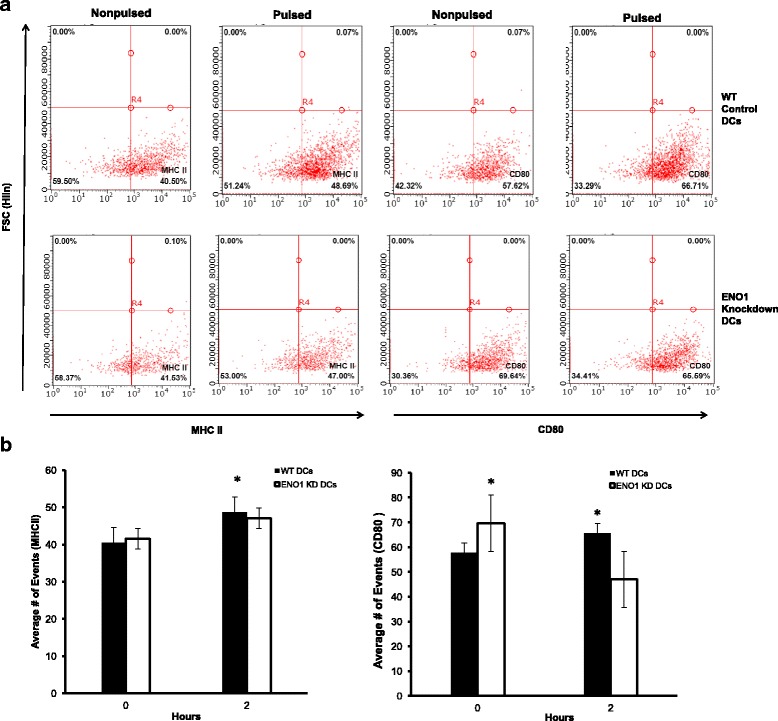
ENO1 regulates DC maturation and activation (2). **a** Flow Cytometry analysis was also used to determine the population of cells with the maturation and activation markers CD80 and MHC II expression in DCs. Cells were stained with FITC conjugated MHC II and CD80. *Green-B-Fluorescence* indicates FITC staining and *Yellow-B Fluorescence* indicates PE staining. **b** The *graph* shows that there were significant differences between WT and ENO1 knockdown DCs (*p* < 0.05). The expression of CD80 and MHC II were lower in Chlamydia pulsed ENO1 knockdown DCs compared to WT DCs. Experiment was repeated 3 times for each sample. * Denotes statistical significance

**Fig. 7:**
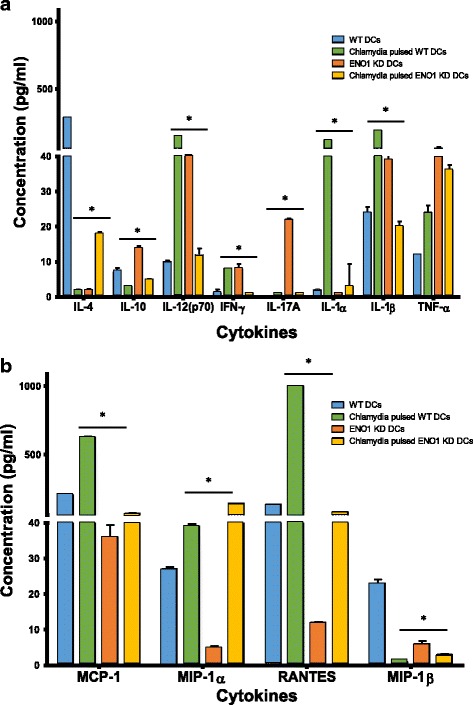
ENO1 regulates DC maturation and activation (3). **a** Bio-Plex Pro Mouse Cytokine 23-Plex multiplex array (Bio-Rad, Hercules, CA) was used for cytokine analysis according to manufacturer’s guidelines. The expression of Th1 type cytokines (IL-1α, IL-1β, IL-12, and IFN-y) were low (*p* ≤ 0.05), while, there was a high expression of the Th2 cytokines IL-4, IL-10, and IL-17 in Chlamydia pulsed ENO1 knockdown DCs compared to Chlamydia pulsed WT DCs (*p* ≤ 0.05). TNF-α was highly expressed in ENO1 knockdown DCs (*p* ≤ 0.05). Experiment was repeated 3 times. **b** Chemokine analysis showed that Chlamydia pulsed ENO1 knockdown DCs have low expression of monocyte chemoattractant protein 1 (MCP-1) and Rantes and high expression of MIP-1α and MIP-1β compared to Chlamydia pulsed WT DCs. * denotes statistical significance. Experiment was repeated 3 times

IL-10 deficient DCs exhibit rapid maturation and activation to efficiently present chlamydial antigens [17]. Therefore recent data revealing that the reprogramming of DC metabolism controls their maturation, activation and antigen presenting functions [14] would suggest that ENO1-mediated modulation of DC metabolism would affect the maturation and activation of DCs. We analyzed the effect of ENO1 knockdown on maturation and activation markers of DCs using flow cytometry. The results showed that TLR4 was significantly lower in all ENO1 knockdown DCs compared to WT DCs (*p* ≤ 0.05) (Fig. 5). CD40 was upregulated in ENO1 knockdown DCs (Fig. 5). In addition, when we analyzed other maturation markers MHC II, CD80 and CD86, the results showed that all three markers had a meaningfully lower expression in *Chlamydia*-pulsed ENO1 knockdown DCs compared to *Chlamydia*-pulsed WT DCs (*p* ≤ 0.05) (Figs. 5 & 6). To further determine the effect of ENO1 on the ability of DCs to activate T cells, we evaluated the cytokine profile of DCs. Results shown that Th1 type cytokine (IL-1α, IL-1β, IL-12, and IFN- γ) production was statistically lower in *Chlamydia*-pulsed ENO1 knockdown DCs (*p* ≤ 0.05), while they concomitantly expressed a higher amount of the CD4^+^ T helper cell type 2 (Th2) cytokines IL-4, IL-10, and IL-17 compared to *Chlamydia*-pulsed WT DCs (*p* ≤ 0.05) (Fig. 7a). Interestingly, tumor necrosis factor (TNF)-α, which induces apoptosis of infiltrating inflammatory cells during genital *Chlamydia* infection [27] was highly expressed in ENO1 knockdown DCs (*p* ≤ 0.05) (Fig. 7a). We also analyzed some chemokines, and the results were mixed, with *Chlamydia*-pulsed ENO1 knockdown DCs having reduced expression of MCP-1 and Rantes and increased expression of macrophage inflammatory proteins (MIP) -1α and -1β compared to *Chlamydia*-pulsed WT DCs (Fig. 7b). Rantes and MCP-1 recruit monocytes, memory T cells, and dendritic cells to the sites of inflammation produced by infection. MIP proteins activate human granulocytes (neutrophils, eosinophils and basophils) too much of which could lead to acute neutrophilic inflammation [28, 29]. Thus overall, the suppression of ENO1 in *Chlamydia-*pulsed DCs appeared to have a deleterious effect on DCs’ activating properties, by producing the inflammatory type of cytokines and chemokines, and enhancing apoptosis.

### Effect of ENO1 on the antigen presenting function of DCs in vivo and in vitro

**Fig. 8:**
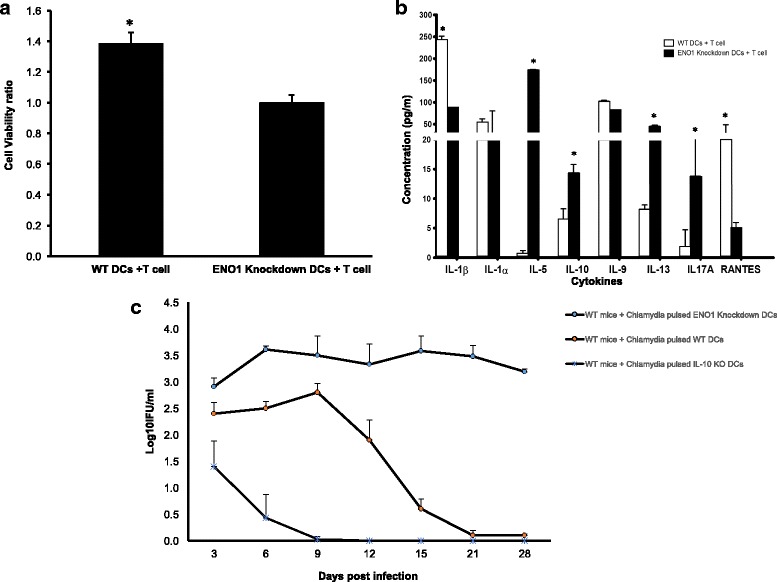
Effect of ENO1 on the antigen presenting function of DCs in vivo and in vitro. **a** The T-cells collected from the spleen of *Chlamydia* infected WT mice were co-cultured with WT and ENO1 knockdown DCs, and their supernatant was collected for cytokine analysis. Proliferation assay was also performed to determine T cell activation. Data showed that ENO1 knockdown DCs had lower levels of antigen specific proliferation of T cells (*p* ≤ 0.05). * Denotes statistical significance. Experiment was repeated 3 times. **b** Results showed that there was a decrease in IL-1α, IL-1β, & Rantes secretion and an increased secretion of IL-5, IL-13, IL-17A, and IL-10 in Chlamydia pulsed ENO1 knockdown DCs incubated with immune CD4+ T cells (*P* ≤ 0.01). * Denotes statistical significance. Experiment was repeated 3 times. **c** We adoptively transferred WT and ENO1 knockdown DCs into naive WT mice through tail vein injection. The mice were then infected intravaginally with 10^5^ IFU of *C. muridarum* 1 week after adoptive transfer. The course of infection and bacterial load were monitored. WT mice that received Chlamydia pulsed ENO1 knockdown DCs experienced a longer period of bacterial shedding compared to the Chlamydia pulsed WT DCs and IL-10^−/−^ (KO). * Denotes statistical significance

IL-10 deficient DCs exhibited efficient chlamydial antigen presentation and activated a high frequency of specific T cells and antibodies against *Chlamydia* [17, 20]. To further determine the role of ENO1 in the antigen presenting function of DCs in vivo and in vitro DCs, which were pulsed with *C. muridarum* for 2 h, were co-cultured with splenic CD4+ T cells from immune mice for 5 days. We compared the antigen presenting function of DCs in WT and ENO1 knockdown DCs. Data showed a significant impairment in the ability of ENO1 knockdown DCs to induce antigen specific proliferation of T cells and cytokine secretion (*p* ≤ 0.05) (Fig. 8a). It was not feasible to include ENO1-over-expressing WT DCs in these studies for technical reasons. Results showed a decrease in secretion of IL-1α, IL-1β, & Rantes and an increase in secretion of IL-5, IL-13, IL17A, and IL-10 in Chlamydia pulsed ENO1 knockdown DCs incubated with immune CD4+ T cells compared to the WT DCs incubated with immune T cells (*P* ≤ 0.01) (Fig. 8b). This result indicates that the absence of ENO1 in DCs abrogates their capacity to present chlamydial antigen to T cells, which will lead to antigen specific Th2 immunity against *Chlamydia* infection in vivo. To further buttress our hypothesis ENO1 knockdown causes a dysregulation of DC metabolism that alters the DC function during Chlamydia infection. Chlamydia pulsed WT, IL-10^−/−^ and ENO1 knockdown DCs were adoptively transferred to naive WT mice though tail vein injection. The mice were then infected intravaginally with 10^5^ IFU of *C. muridarum* 1 week after adoptive transfer. The course of infection and bacterial load were monitored. WT mice that received Chlamydia pulsed ENO1 knockdown DCs experienced a longer period of bacterial shedding and higher bacterial load when compared to the Chlamydia pulsed IL-10^−/−^ and WT DCs (Fig. 8c). In vivo data suggests that ENO1 knockdown DCs lose the ability to induce a robust Th1 immune response that is required for infection clearance.

## Discussion

IL10^−/−^ mice have been shown to clear Chlamydia infections at a faster rate than WT mice, and this protective ability of the IL-10^−/−^ mice has been linked with the presence of IL-10^−/−^ DCs [17, 20]. In our ongoing study of the function of IL-10^−/−^ DCs during Chlamydia infection, we observed using 2-DIGE proteomics, the differential expression of the protein ENO1, which was up regulated in IL-10^−/−^ DCs compared to WT DCs. We later validated this through Western blotting and confocal microscopy analysis, where we showed that ENO1 expression decreased with time in WT DCs during Chlamydia infection, however, the levels of ENO1 in IL-10^−/−^ DCs remained consistently high all through. In this study, we performed a preliminary study to elucidate a role for ENO1 in the function of DCs during Chlamydia infection. ENO1 is a glycolytic enzyme, which has been found to play other roles in inflammation, tumor suppression, monocyte and mast cell differentiation [21, 22]. To understand the role of ENO1 in DCs during chlamydia infection, we first determined its intracellular location, as this had been shown to play a role to play in its function. We determined that ENO1 was mainly located in the cytoplasm, and that this would be related to its primary role in glycolysis, the conversion of 2-phosphoglycerate to phosphoenolpyruvate [22].

To find out if ENO1 was associated with the immunoregulatory properties of DCs during chlamydia infection we first silenced ENO1 gene in DCs using siRNA lentiviral system to knockdown the gene. This knockdown was completed at 48 h after the addition of the virus. We then determined the amount of pyruvate produced in chlamydia pulsed ENO1 knockdown and WT DCs in a time course experiment. We noticed that the amount of pyruvate produced in chlamydia pulsed ENO1 knockdown DCs was significantly lower than that produced by chlamydia pulsed WT DCs. This result was expected since ENO1 was responsible for dehydrating 2-phosphoglycerate to phosphoenolpyruvate which is then converted to pyruvate [22]. It highlighted the fact that the rate of producing pyruvate which is the final product of glycolysis was being hindered in the chlamydia pulsed ENO1 knockdown DCs, which might influence the function of these DCs. Checking to see if the enzyme pyruvate dehydrogenase which is responsible for converting pyruvate to acetyl CoA would be affected by the reduction in the amount of pyruvate produced, we noticed no difference in the expression of pyruvate dehydrogenase between the chlamydia pulsed or nonpulsed WT and ENO1 knockdown DCs. This meant that knockdown of ENO1 in DCs only affects pyruvate production and it does not affect the other enzymes involved in the metabolic process. It is becoming increasingly clear that DC activation and function coincide with cellular metabolism, which is tailored towards the bioenergetic and biosynthetic needs of cells [14, 15]. Notable changes during activation include enhanced glycolysis, the accumulation of succinate and the biosynthesis of fatty acids from citrate to support lipid biosynthesis. Those alterations in metabolism are required to meet the increased biosynthetic and bioenergetic demands of activated DCs specifically by funneling metabolites into pathways for lipid and protein synthesis [14, 30–33]. Disruption of glycolysis through the reduced expression of ENO1 has recently been linked to NLRP3 activation [23], and we have previously shown that NLRP3 inflammasome assembly was suppressed in IL-10^−/−^ DCs [18], which we now know to have abundantly expressed ENO1, this shows an interesting linkage of ENO1 to the inflammasome that would be explored further.

Collectively, these metabolic changes in DCs also facilitate the homeostasis of their mitochondria, which are center hubs for metabolic activity. Therefore, we evaluated the morphology of mitochondria in WT and ENO1 knockdown DCs nonpulsed or pulsed with Chlamydia. We found that the morphology of the mitochondria in the Chlamydia pulsed ENO1 knockdown DCs was markedly different from the regular mitochondrial morphology which was apparent in Chlamydia pulsed WT DCs and even to some extent in the nonpulsed ENO1 knockdown DCs. The mitochondria in Chlamydia pulsed ENO1 knockdown DCs appeared fissed, swollen and had loose cristae, which appeared, shortened, stumpy and dispersed. It seems that the reduced production of pyruvate caused by the silencing of ENO1 has led to a drastic change in mitochondrial structure causing cristae remodeling, which becomes apparent when the DC is pulsed with Chlamydia. This remodeling has been reported recently by another group specializing in effector and memory T cells; where their data indicated that mitochondria cristae remodeling and/or fission acts as a signal to drive the induction of aerobic glycolysis and subsequent cell activation [15], they also validated this in DCs and macrophages. Here we postulate that the Chlamydia pulsed WT mitochondria were still in their tubular fused form and so would function in the oxidative phosphorylation pathway, while the mitochondria in the Chlamydia pulsed ENO1 knockdown DCs were in the fissed form and so were prone to have reduced electron transport chain efficiency and promote aerobic glycolysis. To find out if underlying modifications in the expression of other mitochondrial associated proteins are associated with this change in morphology, we looked at the expression of TOM20 and ANT 1/2/3/4. There was a differential expression of TOM20 and ANT 1/2/3/4 in Chlamydia pulsed ENO1 knockdown DCs compared to Chlamydia pulsed WT DCs. Tom 20 is a central component of the translocase of the outer membrane (TOM) complex receptor, which recognizes and transports mitochondrial preproteins synthesized in the cytosol [34]. ANT is in the inner membrane of the mitochondria and is a component of the permeability transition pore complex, which has been established to play an important role in mitochondrial homeostasis [35]. Cellular models have shown that permeability transition pore complex opening dissipates the transmembrane inner potential, triggers matrix swelling, releasing cytochrome C into the cytosol, and subsequent cell apoptosis. It has been shown in ANT knockout (KO) studies that ANT not only plays an essential structural role but also has a regulatory function for permeability transition pore complex (PTPC) [36]. In this study, TOM20 and ANT1/2/3/4 expression were differentially expressed in Chlamydia pulsed ENO1 knockdown DCs with the mitochondria having progressively enlarged cristae and intermembrane spaces, suggesting that these proteins might be essential in determining the permeability of the mitochondria.

In this study, we have shown that Chlamydia pulsed ENO1 knockdown DCs have dysregulated mitochondria, which appeared swollen, fissed and have shortened/dispersed cristae. We have also shown that there are changes in proteins associated with mitochondria permeability. All these changes should inherently lead to a high level of apoptosis in these DCs. Therefore, we wanted to find out if this influenced DC survival. The results showed that there was an apparent increase in apoptosis in chlamydia pulsed ENO1 knockdown DCs compared to chlamydia pulsed WT DCs. This result implies that the alterations of DC metabolism and the mitochondria did actually have an effect on DC survival [37].

We then hypothesized that these changes in metabolism, mitochondrial structure and survival in chlamydia pulsed ENO1 knockdown DCs would have an impact on the activation and function of these DCs. We decided to look at the DCs maturation and activation markers including the cytokines produced, as well as the antigen presenting function of these DCs. Results show that Chlamydia pulsed ENO1 knockdown DCs had fewer cells expressing TLR4, CD80, CD86 and MHC II compared to Chlamydia pulsed WT DCs, thus implying that ENO1 is integral in maintaining the maturation of DCs. We further assessed the probability that this phenomenon was real, by analyzing the cytokines and chemokines produced by these DCs. There was a lower expression of the Th1 cytokine IL-12, and a higher expression of the Th2 cytokines IL-4 and IL-10 in Chlamydia pulsed ENO1 knockdown DCs compared to Chlamydia pulsed WT DCs. This result could be interpreted to mean that when ENO1 is silenced, the DCs become less inclined to fight off Chlamydia infection, since we know that the immune response against Chlamydia requires a Th1 and not Th2 response. Amongst the cytokines we evaluated, we observed that IFN-γ expression was decreased in Chlamydia pulsed ENO1 knockdown DCs compared to Chlamydia pulsed WT DCs. We know that IFN-γ is an activator of T-bet and an inhibitor of GATA-3, which are transcription factors regulating Th1 and Th2 differentiation respectively [38]. This result implies that IFN-γ would not be available to inhibit GATA-3, thus leading to a tilt towards the Th2 phenotype. The pro-inflammatory cytokines IL-1α and IL-1β were expressed at lower levels in Chlamydia pulsed ENO1 knockdown DCs compared to Chlamydia pulsed WT DCs, however TNF-α another pro-inflammatory cytokine was highly expressed in ENO1 knockdown DCs. We know that having a good boost of early IL-1 production is good for priming the innate immune system [18, 39], thus the reduced production of IL-1 in Chlamydia pulsed ENO1 knockdown DCs constitutes an immune deficiency in the clearance of Chlamydia. Increased production of TNF-α has been associated with inflammatory conditions that might lead to pathogenesis of the local area of infection [40]. In addition to the cytokine we also studied the chemokine produced by the DCs. We had mixed results with Chlamydia pulsed ENO1 knockdown DCs having reduced expression of MCP-1 and Rantes and increased expression of MIP-1α and MIP-1β compared to Chlamydia pulse WT DCs. It has been shown MCP-1 and Rantes are important in signaling cells of the innate immune and adaptive immunity to migrate towards the site of infection [28, 29]. In a bid to further determine the role of ENO1 in activation of T cells we performed T cell proliferation assays and the results showed that T cell proliferation was significantly lower in ENO1 knockdown DCs compared to WT DCs, in addition, cytokine assay results showed that Th2 enhancing cytokines IL-5, IL-10 and IL-13 expression, were increased in cultures with Chlamydia pulsed ENO1 knockdown DCs. This result further buttresses the fact that Chlamydia pulsed ENO1 knockdown DCs were more inclined to balance the immune system towards the Th2 phenotype, which would be unable to clear a Chlamydia infection. This is in addition to the fact that the expression of the chemokine Rantes was also lowered in the co-cultures with Chlamydia pulsed ENO1 knockdown DCs. This would lead to a reduction in the migration of cells to the point of infection. Our results has been corroborated by some recent studies that have established that ENO1 is able to activate CD14-dependent TLR4 pathway on monocytes, which involves a dual mechanism firstly pro-inflammatory and secondly anti-inflammatory [41]; and that ENO1 has also been linked to the regulation of differentiation of mast cells [42].

Furthermore, we wanted to show in vivo that ENO1 is important in clearing Chlamydia infection, so we carried out an adoptive transfer experiment. The results showed that WT mice that were adoptively transferred with Chlamydia pulsed IL-10^−/−^ and WT DCs were better able to clear their Chlamydia infection compared to mice that were adoptively transferred with Chlamydia pulsed ENO1 knockdown DCs. This consolidated our previous results showing that the Chlamydia pulsed ENO1 knockdown DCs were not able to function optimally in clearing the Chlamydia in the mice because of the absence of ENO1, which we believe is regulated by IL-10. This result has implication in the use of ENO1 as a therapeutic treatment during Chlamydia infection; in addition, it has been shown that ENO1 deoxyribonucleic acid elicits humoral and cellular immune responses against pancreatic tumors, delays tumor progression [43].

## Conclusions

In summary, we have concluded that ENO1 is important in the functioning of DCs during chlamydia infection. We first observed this possibility by the high levels of ENO1 produced in IL-10^−/−^ DCs, and we were inclined to find out if indeed this phenomenon had a real bearing in the Chlamydia clearing ability of IL-10^−/−^ DCs. Results showed that the reduction in ENO1 expression in WT DCs happened after a period and that this led to a reduction in pyruvate which we believe is central to all the various factors involved in the process of metabolism and the eventual maturation and activation of the DCs. New studies show that immune cell activation coincides with cellular metabolism that is tailored towards the bioenergetic and biosynthetic needs of these cells. Studies have demonstrated the contribution of cellular metabolic pathways to the ability of immune cells to sense the microenvironment and to alter their function [12–15]. Changes in the microenvironment induce a broad spectrum of inducible and reversible metabolic programs, which in turn forms the basis of the inducible and reversible spectrum of functional immune cell activation/polarization phenotypes. This alteration in glycolytic metabolism and mitochondrial morphology, which we have observed in this study, might be required to meet the increased biosynthetic and bioenergetic demands of the Chlamydia, activated DCs [14]. The findings in this study suggest that ENO1 can be used as an immunotherapeutic strategy for inducing adequate and long-term immunity against Chlamydia-induced tubal pathologies. This because there are presently no Food and Drug Administration (FDA) approved, commercially available vaccines with built-in biologically safe immunomodulators, so if successful, our findings could have important implications for the rational design of next-generation vaccines for boosting vaccine efficacy. Modulation of alpha enolase thus provides a potentially effective strategy to boost DC function and promote immunity against intracellular microbial pathogens.

## Abbreviations

2D-DIGE: Two-dimensional fluorescence differential gel electrophoresis analysis
7-AAD: 7-amino-actinomycin D
ANT: Adenine nucleotide translocator
APC: Allophycocyanin
CD: Cluster of differentiation
DCs: Dendritic cells
EBs: Elementary bodies
ENO1: Alpha enolase
FDA: Food and Drug Administration
FITC: Fluorescein isothiocyanate
GM-CSF: Granulocyte-macrophage colony-stimulating factor
IFN-γ: Interferon gamma
IFU: Inclusion forming units
IL: Interleukin
IL-10^−/−^: IL-10 knockout
LEK1: Leucine/glutamic acid/lysine protein 1
MCP-1: Monocyte chemoattractant protein 1
MEM: Minimum essential medium
MHC: Major histocompatibility complex
MIP-1α: Macrophage Inflammatory Protein -alpha
MIP-1β: Macrophage Inflammatory Protein -beta
NLRP3: NLR family pyrin domain containing 3
OXPHOS: Oxidative phosphorylation
PE: Phycoerythrin
PTPC: Permeability transition pore complex
TEM: Transmission electron microscopy
TFI: Tubal factor infertility
Th1: CD4^+^ T helper cell type 1
Th2: CD4^+^ T helper cell type 2
TLR4: Toll like receptor 4
TNF-α: Tumor necrosis factor- alpha
WT: Wild type
